# The Effects of Mammary Gland *ATIII* Overexpression on the General Health of Dairy Goats and Their Anti-Inflammatory Response to LPS Stimulation

**DOI:** 10.3390/ijms242015303

**Published:** 2023-10-18

**Authors:** Laiqing Yan, Hao Wu, Shengyu Guan, Wenkui Ma, Yao Fu, Pengyun Ji, Zhengxing Lian, Lu Zhang, Yiming Xing, Bingyuan Wang, Guoshi Liu

**Affiliations:** 1 State Key Laboratory of Animal Biotech Breeding, National Engineering Laboratory for Animal Breeding, Key Laboratory of Animal Genetics and Breeding of the Ministry of Agriculture, Beijing Key Laboratory of Animal Genetic Improvement, College of Animal Science and Technology, China Agricultural University, Beijing 100193, China; laiqingyan@cau.edu.cn (L.Y.); 18800160525@163.com (H.W.); gsy729@cau.edu.cn (S.G.); ma17610890927@163.com (W.M.); fuyao2019@cau.edu.cn (Y.F.); pengyunji@cau.edu.cn (P.J.); lianzhx@cau.edu.cn (Z.L.); luzhang2018@cau.edu.cn (L.Z.); 2 State Key Laboratory of Agrobiotechnology, College of Biological Sciences, China Agricultural University, Beijing 100193, China; ymxing@cau.edu.cn

**Keywords:** antithrombin III, transgenic cloned dairy goat, inflammation, milk quality, intestinal microorganism

## Abstract

Antithrombin III is an important anticoagulant factor with anti-inflammatory properties. However, few studies have explored its anti-inflammatory actions in *ATIII* overexpressed transgenic animals. In this study, the dairy goats with mammary overexpression of *ATIII* were used to investigate their general health, milk quality and particularly their response to inflammatory challenge. The results showed that transgenic goats have a normal phenotype regarding their physiological and biochemical parameters, including whole blood cells, serum protein levels, total cholesterol, urea nitrogen, uric acid, and total bilirubin, compared to the WT. In addition, the quality of milk also improved in transgenic animals compared to the WT, as indicated by the increased milk fat and dry matter content and the reduced somatic cell numbers. Under the stimulation of an LPS injection, the transgenic goats had elevated contents of IGA, IGM and superoxide dismutase SOD, and had reduced proinflammatory cytokine release, including IL-6, TNF-α and IFN-β. A 16S rDNA sequencing analysis also showed that the transgenic animals had a similar compositions of gut microbiota to the WT goats under the stimulation of LPS injections. Mammary gland *ATIII* overexpression in dairy goats is a safe process, and it did not jeopardize the general health of the transgenic animals; moreover, the compositions of their gut microbiota also improved with the milk quality. The LPS stimulation study suggests that the increased *ATIII* expression may directly or indirectly suppress the inflammatory response to increase the resistance of transgenic animals to pathogen invasion. This will be explored in future studies.

## 1. Introduction

Antithrombin III (ATIII), encoded by the SerpinC1 gene, is a serine protease inhibitor produced in the process of coagulation cascade. ATIII inhibits the action of proteases by interacting with heparin-like substances on the surface of endothelial cells. It can also inhibit other procoagulant factors including thrombin and factors IXa, Xa, XIa and XIIa, as well as FVIIa [1,2]. As an important anticoagulant factor, approximately 60–70% of the body’s antithrombin activity is carried out by this factor [3]. It also has a strong anti-inflammatory effect, which is mainly mediated by its anticoagulant effect [4,5], while some studies indicate that its anti-inflammatory effect may be achieved by increasing prostacyclin (PGI2) [6,7]. Another study reported that the expression of IL-6 and IL-8 in monocytes was significantly down-regulated by ATIII, suggesting that the anti-inflammatory properties of ATIII may also mediated by its action on monocytes [3]. Its application significantly reduced heat stroke inflammation injuries of various organs in a rat model, and improve the survival rate [8].

Generally, pathogen infection will initiate the innate immune response, which can trigger blood coagulation [9]. The components involved in the thrombin process are not simply bystanders, but can either amplify or inhibit the inflammatory response, depending on the physiopathological status. On other hand, inflammatory cytokines and endotoxin can induce the de novo synthesis of tissue factor in leukocytes to initiate blood clotting [10]. Therefore, ATIII may have potential therapeutic applications in critically ill patients and in the settings of perioperative anticoagulation management for surgery and obstetrics related to inflammation [5]. Systemic inflammatory response syndrome (SIRS) and sepsis are associated with high morbidity and mortality [11,12]. They are usually caused by Gram-negative bacteria infection; the lipopolysaccharide (LPS) from the membranes of these bacteria binds to CD14 receptors and activates the inflammatory cascade. LPS can also bind to soluble CD14 to activate endothelial cells and smooth muscle cells, eventually leading to systemic inflammatory response syndrome and sepsis [13]. In sepsis, the diffuse activation of the coagulation system and its exhaustion lead to disseminated intravascular coagulation (DIC), resulting in organ dysfunction and organ failure. One of the main participants in this physiopathological process is *ATIII* [14]. Studies have shown that *ATIII* levels in patients with sepsis decrease due to its decreased synthesis and increased consumption [15]. LPS is also the main pathogenic factor of mastitis in dairy cattle [16]. It promotes the release of inflammatory cytokines and oxidative mediators [17]. Inhibition of inflammation and oxidative stress can reduce the severity of mastitis [18]. ATIII exhibits anti-inflammatory effects, and an increase in its level in animals, particularly in dairy goats, may have therapeutic effect on their mastitis. One study has reported the expression of recombinant human antithrombin ⅲ in goat mammary glands, and has suggested the product of ATIII could be used therapeutically for the purpose of anticoagulation [19]. 

However, research on the function of *ATIII* has mostly focused on cell culture or small animals such as mice, and few studies have dealt with its anti-inflammatory effects, especially endogenously produced ATIII. To fill this gap, in the current study, using transgenic animals, we first evaluated whether mammary gland overexpression of *ATIII* in dairy goats would influence their normal physiology and their milk production and quality. Then, these animals were challenged with an LPS injection to investigate their inflammatory response compared to the WT (Wild type). The results enable us to better understand the role of *ATIII* in the process of inflammation in the body. 

## 2. Results

### 2.1. Effects of Mammary Gland ATIII Overexpression in Dairy Goats on Blood Cells under Normal Conditions

**Figure 1:**
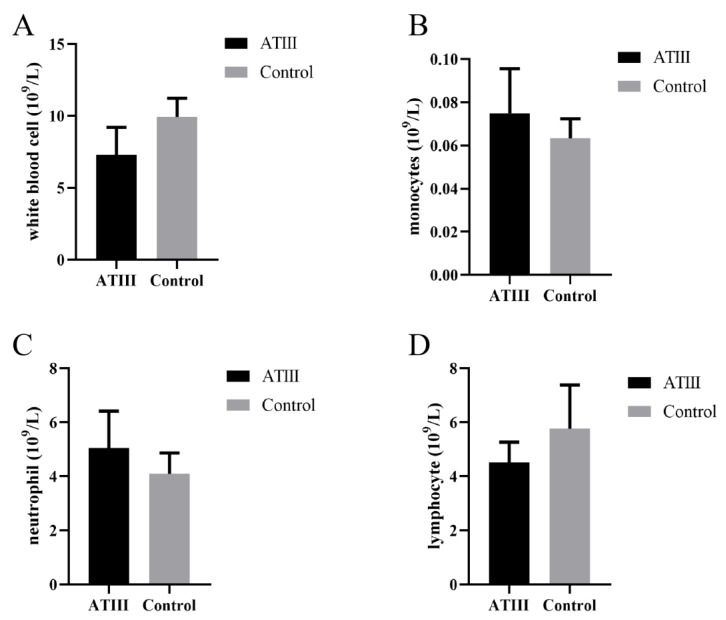
Effects of mammary gland ATIII overexpression in dairy goats on blood cells under normal conditions. (**A**) leukocytes; (**B**) monocytes; (**C**) neutrophils; (**D**) lymphocytes. ATIII: mammary gland ATIII overexpression, (n = 6).

In dairy goats overexpressing ATIII in their mammary glands, effects on their blood cells were evaluated. The results showed that under normal conditions, there was no significant difference in leukocytes, neutrophils, monocytes, and lymphocytes between dairy goats overexpressing ATIII in their mammary glands compared to WT goats (Figure 1).

### 2.2. Effects of Mammary Gland ATIII Overexpression in Dairy Goats on Erythrocytes under Normal Conditions

**Figure 2:**
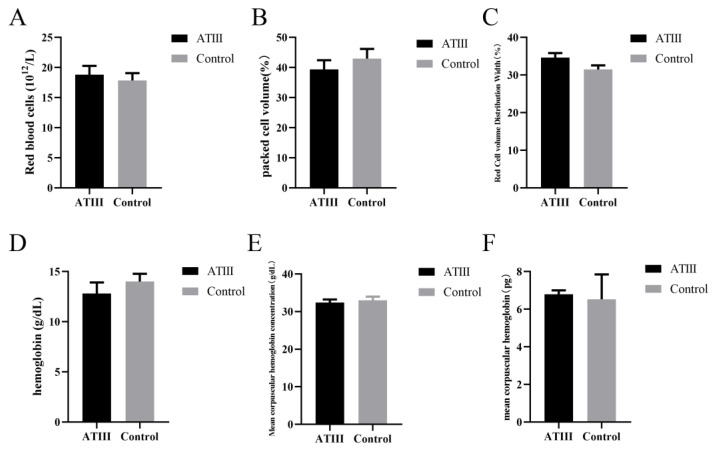
Effects of mammary gland ATIII overexpression in dairy goats on erythrocytes under normal conditions. (**A**) erythrocyte content; (**B**) hematocrit; (**C**) erythrocyte width; (**D**) hemoglobin; (**E**) average hemoglobin concentration; (**F**) average erythrocyte hemoglobin concentration. ATIII: mammary gland ATIII overexpression, (n = 6).

The results showed that under normal conditions, there was no significant difference in the erythrocyte index between mammary gland *ATIII*-overexpressing dairy goats and WT goats (Figure 2).

### 2.3. Effects of Mammary Gland ATIII Overexpression in Dairy Goats on the Biochemical Parameters of Blood under Normal Conditions

**Figure 3:**
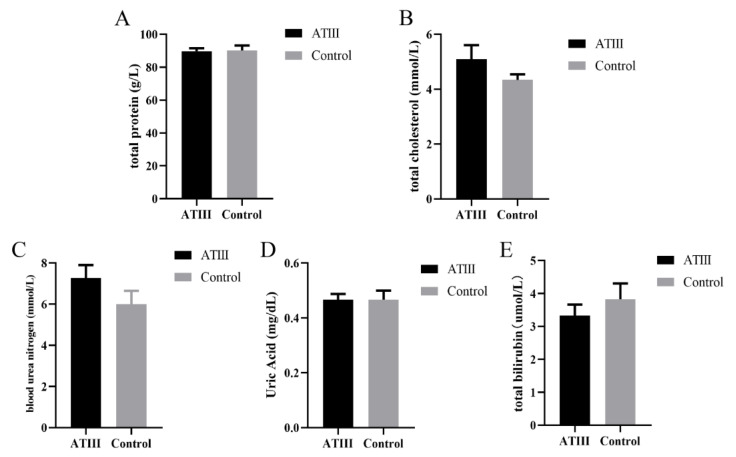
Effects of mammary gland ATIII overexpression in dairy goats on blood biochemical parameters under normal conditions. (**A**) total protein; (**B**) total cholesterol; (**C**) urea nitrogen; (**D**) uric acid; (**E**) total bilirubin. ATIII: mammary gland ATIII overexpression, (n = 6).

The results showed that there were no significant differences in total protein, total cholesterol, urea nitrogen, uric acid, and total bilirubin between mammary gland *ATIII*-overexpressing dairy goats and WT goats (Figure 3).

### 2.4. Effects of Mammary Gland ATIII Overexpression in Dairy Goats on Dairy Herd Improvement (DHI) under Normal Conditions

**Figure 4:**
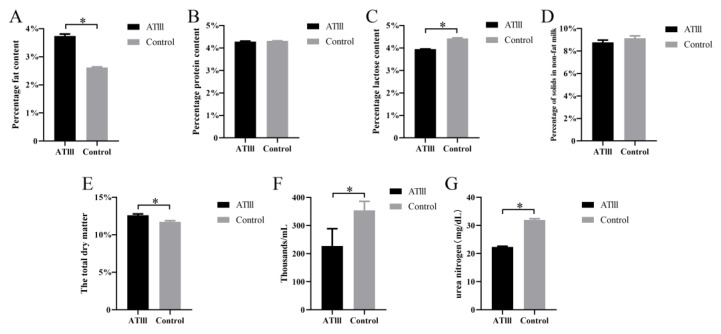
Effects of mammary gland ATIII overexpression in dairy goats on the quality of milk under normal conditions. (**A**) milk fat; (**B**) milk protein; (**C**) lactose; (**D**) non-fat milk solid; (**E**) dry matter; (**F**) somatic cell count; (**G**) urea nitrogen. ATIII: mammary gland ATIII overexpression, (n = 6) * *p* < 0.05.

DHI is an important parameter used to evaluate the milk quality. The results showed that the contents of milk fat and dry matter in the milk in dairy goats with mammary gland ATIII overexpression were significantly higher than those of WT goats. In contrast, the contents of lactose, urea nitrogen and somatic cells in the milk of mammary gland ATIII-overexpressing dairy goats were significantly lower than those of WT goats. There were no significant differences in the content of milk protein and non-fat milk solids between the groups. The results indicated that the milk quality of dairy goats with mammary gland ATIII overexpression was improved compared to the WT goats (Figure 4).

### 2.5. Effects of Mammary Gland ATIII Overexpression in Dairy Goats on Immunoglobins and SOD under LPS Stimulation

**Figure 5:**
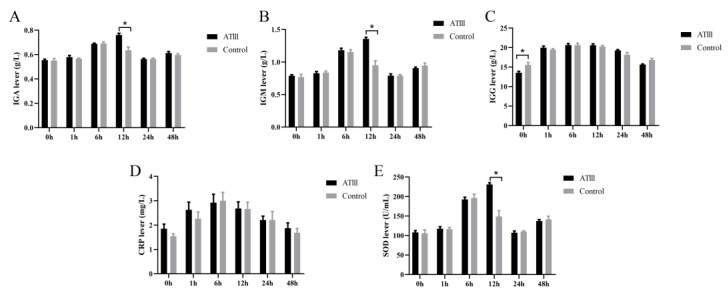
Effects of mammary gland ATIII overexpression in dairy goats on immunoglobins and SOD under LPS stimulation. (**A**) IGA; (**B**) IGM; (**C**) IGG; (**D**) CRP; (**E**) SOD. ATIII: mammary gland ATIII overexpression n = 6, * *p* < 0.05.

The results showed that C-reactive protein significantly increased within 1 h after LPS injection; the peak was at 6 h after injection, and the level then slowly returned to pre-injection levels, and no significant difference in this parameter was observed between the groups. The levels of IGA, IGM, IGG and other immunoglobulins also significantly increased after LPS injection, and their peaks appeared at 12 h after injection. Importantly, at 12 h after LPS injection, the levels of these immunoglobulins were significantly higher in goats with mammary gland ATIII overexpression than those in WT goats. Interestingly, at 0 h (before LPS injection), the IGG content of mammary gland ATIII-overexpressing dairy goats was lower than that of the WT group. The SOD level exhibited similar changes to the immunoglobins (Figure 5).

### 2.6. Effects of Mammary Gland ATIII Overexpression in Dairy Goats on Pro-Inflammatory Factors after LPS Stimulation

**Figure 6:**
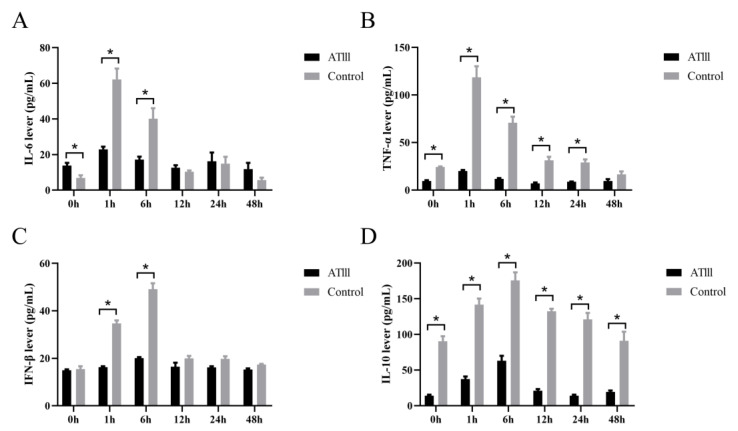
Effects of mammary gland ATIII overexpression in dairy goats on of the pro-inflammatory factors (**A**) IL-6; (**B**) TNF-α; (**C**) IFN-β; (**D**) IL-10. ATIII: mammary gland ATIII overexpression, n = 6, * *p* < 0.05.

Regarding IL-6, baseline levels (0 h) in transgenic goats were significantly higher than that in the WT. After LPS injection, the levels significantly increased in both groups and reached a peak at 1 h after injection; they returned to baseline 12 h after injection. At 1 and 6 h after the injection, the IL-6 levels in WT goats were significantly higher than that in goats with mammary gland ATIII overexpression (Figure 6A). Regarding IL-10, levels significantly increased after LPS injection, with a peak at 6 h, and gradually reduced to baseline levels at 48 h after injection. However, levels were significantly higher in the WT goats than that in the transgenic goats at all the time points tested (Figure 6D). The TNF-α level had a similar tendency to IL-10 (Figure 6B). For IFN-β, no significant difference between the groups was detected at baseline. After the LPS injection, the IFN-β level significantly increased at 1 and 6 h only in the WT goats, and the IFN-β level in transgenic goats remained at the baseline level (Figure 6C).

### 2.7. Effects of Mammary Gland ATIII Overexpression in Dairy Goats on Ear Local Inflammation in after LPS Stimulation

**Figure 7:**
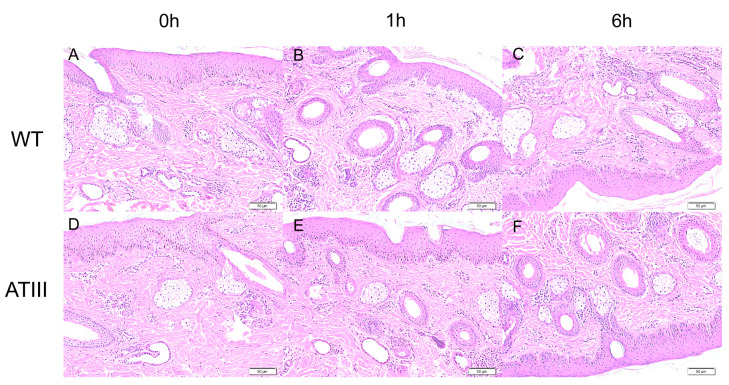
Effects of LPS local injection on ear inflammation on representative histography of ear tissue stained with HE. (**A**–**C**) are tissues from transgenic goats. (**D**–**F**) are tissues from wild-type goats. ATIII: mammary gland ATIII overexpression, WT: wild-type goat, bars = 50 μm.

Since the LPS was injected from the ear, we collected the ear tissues to investigate whether LPS would induce local information. The results showed that there was no significant difference in inflammatory response in goat ear slices before and after LPS injection in both groups, as indicated by the lack of serious inflammatory cell infiltration around the vascular dermis (Figure 7).

### 2.8. Effects of LPS Injection on Intestinal Microflora of Dairy Goats with/without Mammary Gland ATIII Overexpression

**Figure 8:**
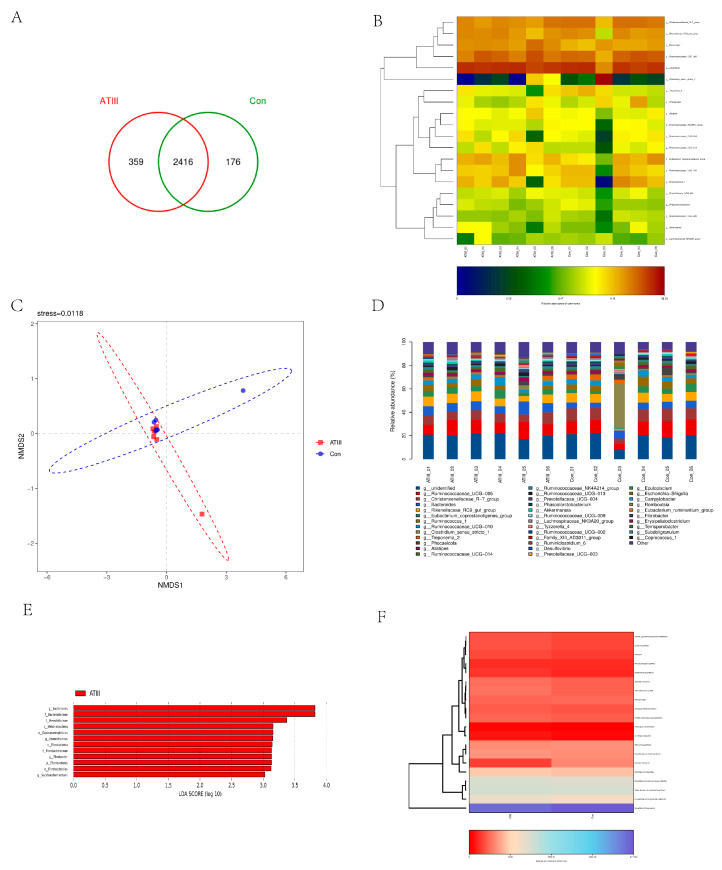
Effects of LPS injection on the intestinal microflora of goats with/out mammary gland ATIII overexpression; (**A**) Wayne diagram; (**B**) OTU and its taxonomic level heatmap chart; (**C**) horizontal species composition analysis histogram; (**D**) partial least-square discriminant analysis based on OTU; (**E**) LDA distribution histogram of LEfSe analysis based on classification information. Note: species with an LDAscore greater than 3 are species with significant differences in abundance in different groups; the length of the histogram represents the influence of significant differences in species. (**F**) PICRUSt-predicted differential path heat map.

In order to explore the effect of LPS on the structure of intestinal microflora, feces were collected for 16S rDNA sequencing. A total of 2951 operational taxonomic units (OTUs) were identified by sequencing, of which 535 were differential OTUs (Figure 8A). A cluster analysis of the OTUs’ taxon at the genus level showed no significant difference between the groups except in Clostridium_sensu_stricto_1. The microflora structure of sample 3 in the WT group was significantly different from those of other samples (Figure 8B). The species composition analysis showed no significant difference in microflora at the genus level among each sample. The top 10 species are g_unidentified, g_Ruminococcaceae_UCG-005, g_Christensenellaceae_R-7_group, g_Bacteroides, g_Rikenellaceae_RC9_gut_group, g_Eubacterium_coprostanoligenes_group, g_Ruminococcus_1, g_Ruminococcaceae_UCG-010, and g_Clostridium_sensu_stricto_1, and there was no significant difference among the samples. There was only some degree of individual difference, For example, content of g-Romboutsia in sample 3 was significantly higher than that of other individuals (Figure 8C). A non-metric multidimensional scaling (NMDS) analysis showed no significant difference in microbial composition between the groups. When the LDA score was set to 3, the lda effect size analysis identified 12 different microflora (g__Bacteroides, f__Bacteroidaceae, f__Prevotellaceae, c__Melainabacteria, o__Gastranaerophilales, g__Anaerotruncus, c__Fibrobacteria, f__Fibrobacteraceae, g__Fibrobacter, p__Fibrobacteres, o__Fibrobacterales, and g__Saccharofermentans) (Figure 8E) in the WT group, of which g_Bacteroides and f_Bacteroidaceae were the most obvious. In addition, the function of the microbial community was analyzed using PICRUSt. A KEGG analysis showed that the differences in the structural and functional pathways of fecal microflora between the two groups were mainly concentrated in the Bacterialchemotaxis pathway (Figure 8F), and no other pathways showed a significant difference. The results confirmed that the overall structure of the intestinal microflora showed no significant difference between mammary gland ATIII-overexpressing goats and WT goats after LPS injection (Figure 8A–E).

## 3. Discussion

Transgenic technology is not only used in biomedical research, but also used in breeding of agricultural livestock [19]. Prokaryotic embryo microinjection and somatic nuclear transfer are technologies most frequently used to generate transgenic livestock [20]. A variety of transgenic livestock have been used as animal models for biomedical research [21]. As an example, many valuable therapeutic proteins are obtained from different transgenic animal models including goats, sheep, cows, pigs, and rabbits [22,23,24,25].

*ATIII* is a critical anti coagulation factor. Approximately 60–70% of the body’s antithrombin activity is carried by this factor [3]. In the current study, transgenic goats with mammary gland *ATIII* overexpression were used (the process was published elsewhere [26]) to investigate the consequences of this process on the general health and the potential anti-inflammatory action of transgenic animals. 

First, we investigated whether this genetic modification would impact the general health and the milk quality of dairy goats. The results showed that this transgenic modification did not modify the blood physiological and biochemical parameters including whole blood cells, total protein, cholesterol, urea nitrogen, uric acid, and total bilirubin. The data indicated the safety of this process for the general health of transgenic animals. In addition, mammary gland *ATIII* overexpression significantly increased the quality of the milk, with increased milk fat and dry matter and decreased lactose, urea, and nitrogen, especially in somatic cells compared with WT goats. 

In addition to these general bio-physiological parameters, we also investigated the potential influences of this transgenic modification on the immunoactivity and inflammatory reactions of the goats, as we know that immunoglobulins play an important role in a variety of physiological processes such as resistance to infection and immune regulation. IgA is an antibody secreted on the surface of the barrier, and its secretion can reach large quantities of more than 1 g per day. This antibody no only prevents pathogen invasion, but also regulates the composition of gut microflora, their gene expression, and metabolism [27]. Here, we found significantly increased levels of IgA and IgM in dairy goats with mammary gland *ATIII* overexpression after LPS stimulation, compared to the WT. The increase in IgA indicates the elevated resistance against pathogens in transgenic goats compared to WT goats. Marianne Boes et al. found that natural IgM played a key role in the immediate prevention of severe bacterial infections [28]. In this study, the IGM levels of the transgenic goats also significantly increased after LPS stimulation compared to those of WT goats. This confirmed the increased pathogen resistance of the transgenic goats. 

It has been reported that *ATIII* has an anti-inflammatory effect, which is related to its inhibition of coagulation cascade reactions [4,5]. Meanwhile, more evidence also indicated that some anti-inflammatory effects of *ATIII* were independent of its anti-coagulation activity [4,29,30,31,32]. The mechanism of this effect is as follows: *ATIII* induces endothelial cells to release prostacyclin, which inhibits platelet aggregation and activation, prevents neutrophils from adhering to blood vessel walls, and reduces various cytokines and chemokines generated by endothelial cells [33,34]. In addition, *ATIII* can directly act on leukocytes and lymphocytes to produce its anti-inflammatory effect [4,29,35]. *ATIII* can inhibit pro-inflammatory cytokines including IL-6 and IL-8 [5]. In this study, it was found that after LPS stimulation, IL-6, TNF-α, and IFN-β levels in transgenic goats were significantly lower than that in WT goats. This is consistent with the previous reports mentioned above. The results indicated a reduced inflammatory response in transgenic goats after LPS stimulation compared to the WT, showing an anti-inflammatory ability. The results are similar to those previously reported, in which administration of ATIII suppressed inflammatory response and reduced mortality in animal models of sepsis and acute pancreatitis [36]. If these observations can apply to clinical situations, administration of ATIII to sepsis patients will open a new avenue for the treatment of this devastating disease. 

Currently, many studies have reported the close association between inflammation, autoimmune diseases, and intestinal microbiota; some studies have reported that antithrombin exhibited anti-inflammatory effects by inhibiting inflammatory cytokine release [37,38]. Yassene Mohammed et al. reported that the intestinal microbiome may also affect the production of thrombin in human subjects [39]. Therefore, we investigated the potential effects of LPS stimulation on the gut microbiota in mammary gland ATⅢ-overexpressing and WT goats with 16S rDNA sequencing. The results showed that, excepting individual variations, the compositions of the gut microbiota and their metabolisms showed no significant differences between the groups. We have also realized a limitation of the study, i.e., the relatively small size of the samples (n = 6 in each group). The reasons for this were two: (1) the remarkably high cost of the transgenic animals; and (2) the power analysis, which suggested that in large animal studies, five animals per group could satisfy the minimal sample size requirement. Considering these two factors, we selected six animals for each group, but this could be improved upon in the future studies. 

In conclusion, mammary gland overexpression of *ATⅢ* in dairy goats not only maintained a healthy milk quality but promoted the milk quality of the transgenic animals compared to the WT. Importantly, under the LPS challenge, the transgenic dairy goats exhibited increased immune response and reduced inflammatory reaction. Currently, the exact mechanisms of these alterations induced by mammary gland overexpression of ATⅢ in dairy goats are unknown. To identify the mechanisms is the goal of our future studies. 

## 4. Materials and Methods

### 4.1. Experimental Animals

Six dairy goats of mammary gland *ATIII* overexpression and six wild-type (WT) dairy goats were sourced from Fengcheng Experimental Base, Jimo District, Qingdao Senmiao Industrial Co., Ltd. (Qingdao, China). The construction method for transgenic goats was published elsewhere [26]. All the experimental goats are Laoshan milk goats of about 3 years old, healthy and with no history of mastitis. All the experimental goats were raised in the same environment. The animals were allowed access to food and water ad libitum. All the treatments of experimental animals involved in this study were approved by the Animal Welfare Committee of China Agricultural University (AW31203202-1-3).

### 4.2. Experimental Design

Goats were divided into two groups: wild-type (WT) (n = 6) and transgenic groups (n = 6). The whole blood cells and biochemical parameters of the animals were measured at the beginning of the study. Then, 100 μL of LPS (normal saline was used as a solvent, and the final concentration was 1 mg/mL) was injected subcutaneously into the ears of the goats. The blood was collected at 0, 1, 6, 12, 24 and 48 h, respectively, to measure the immunoglobulin (IGA, IGM, IGG, CRP, SOD) and inflammatory factors (IL-6, TNF-α, IL-10, IFN-β). At the same time, goat ear tissues and feces were collected for section observation and microbial sequencing analysis, respectively.

### 4.3. Analysis of Blood Cells and Blood Biochemical Parameters 

Briefly, 5 mL blood was collected from the goats’ jugular vein at 0, 1, 6, 12, 24 and 48 h, respectively, after LPS injection. The blood was placed at 37 °C for 30 min and centrifuged at 3000 rpm for 10 min, and the biochemical parameters (total protein, total cholesterol, urea nitrogen, uric acid, total bilirubin) were detected directly with serum. The content of immunoglobulin (Glob) was determined using an automatic dry biochemical analyzer (FDCNX500iVC, Fuji, Japan). The whole blood cells were measured using an automatic hematology analyzer (Sysmex Kmur1000D Magi Sysmex Inc., Kobe, Japan). The numbers of neutrophils (Neu), lymphocytes (Lym), monocytes (Mono), and mononuclear macrophages were recorded. C-reactive protein was determined using a hypersensitive C-reactive protein kit (B2072). The sample detection procedure was carried out according to the manufacturer’s instructions. All samples were tested in the Animal Hospital of Qingdao Agricultural University.

### 4.4. Determination of Milk Composition

Then, 5 mL of milk was collected at 04:00 and 16:00, respectively, and stored at −20 °C until analysis. Milk samples were determined in the national standard material preparation laboratory for the determination of production performance. The DHI index (milk fat, milk protein, lactose, non-fat milk solid, dry matter, somatic cell count, urea nitrogen) was measured using a Milkscan FT + Fossomatic TMFC milk composition and somatic cell count automatic analyzer (Foss Group, Hillerød, Denmark).

### 4.5. Assays of Proinflammatory Cytokines

The levels of inflammatory cytokines in serum were detected using an ELISA (Beijing, China) according to the manufacturer’s instructions. The kits for each cytokine are as follows: IL6[interleukin, 6], eBioscience, 88-7066; IL10[interleukin, 8], eBioscience, 88-8086; IFN-β[Interferon β], eBioscience, 88-7126; and TNF-α[tumor necrosis factor-α], eBioscience, 88-7346.

### 4.6. Hematoxylin-Eosin Staining

The goat ear tissue was fixed with 4% paraformaldehyde and trimmed as serial sections of 4 μm, then embedded in paraffin. The paraffin sections were dewaxed with xylene, dewaxed with gradient alcohol, and finally placed in distilled water. The samples were stained with hematoxylin dye for 20 min, washed with distilled water three times, and separated with 1% hydrochloric acid and alcohol for 5–10 s. Then, the samples went through a blue reaction with 100 mmol/L pH 7.4 PBS for 15 min, before being dehydrated with 70% alcohol and 80% alcohol, respectively, for 2 min each. Finally, the samples were stained with Eosin dye for 1 min and dehydrated again with 95% and 100% alcohol, respectively. The samples were soaked with xylene and sealed with neutral gum for analysis. 

### 4.7. Fecal Samples’ 16S rDNA Sequencing

The fecal samples were collected from the rectum of goats. Briefly, the goats were restrained to limit their movement. The anus of the goat was cleaned with water and a small amount of Vaseline was smeared around the anus. The feces were collected from the rectum using figures covered with aseptic gloves. The fecal samples were immediately stored in containers with dry ice and transported to Jinweizhi Biotechnology Co., Ltd. (Suzhou, China) for 16 s microbial flora sequencing.

### 4.8. Statistical Analysis 

The data are expressed as means ± standard deviation. One-way variance and two-factor variance (ANOVA) analyses were performed, and then Duncan’s test was carried out to compare the differences between the interested groups using SPSS software version 25.0 (IBM SPSS Statistics, Armonk, NY, USA). *p <* 0.05 was considered to be statistically significant.

## 5. Conclusions

All these observations indicated that mammary gland *ATIII* overexpression in dairy goats is a safe process, and it did not jeopardize the general health of the transgenic animals or the composition of their gut microbiota. In addition, it improved the quality of milk by reducing milk somatic cells and the increasing milk fat and dry matter contents compared to WT goats. The LPS stimulation study suggests that the increased *ATIII* expression may directly or indirectly suppress the inflammatory response, and may therefore increase the resistance of transgenic animals to pathogenic invasion. This will be explored in our future studies. 

## Data Availability

The raw data supporting the conclusions of this article will be made available by the authors, without undue reservation.

## References

[1] Rosenberg R.D. (1989). Biochemistry of heparin antithrombin interactions, and the physiologic role of this natural anticoagulant mechanism. Am. J. Med..

[2] Souter P.J., Thomas S., Hubbard A.R., Poole S., Römisch J., Gray E. (2001). Antithrombin inhibits lipopolysaccharide-induced tissue factor and interleukin-6 production by mononuclear cells, human umbilical vein endothelial cells, and whole blood. Crit. Care Med..

[3] Menache D. (1991). Antithrombin III: Introduction. Semin. Hematol..

[4] Horie S., Ishii H., Kazama M. (1990). Heparin-like glycosaminoglycan is a receptor for antithrombin III-dependent but not for thrombin-dependent prostacyclin production in human endothelial cells. Thromb. Res..

[5] Levy J.H., Sniecinski R.M., Welsby I.J., Levi M. (2016). Antithrombin: Anti-inflammatory properties and clinical applications. Thromb. Haemost..

[6] Kellner P., Nestler F., Leimert A., Bucher M., Czeslick E., Sablotzki A., Raspè C. (2014). Antithrombin III, but not C1 esterase inhibitor reduces inflammatory response in lipopolysaccharide-stimulated human monocytes in an ex-vivo whole blood setting. Cytokine.

[7] Lu Z., Wang F., Liang M. (2017). SerpinC1/Antithrombin III in kidney-related diseases. Clin. Sci..

[8] Hagiwara S., Iwasaka H., Shingu C., Matsumoto S., Uchida T., Noguchi T. (2010). High-dose antithrombin III prevents heat stroke by attenuating systemic inflammation in rats. Inflamm. Res..

[9] Opal S.M., Garber G.E., LaRosa S.P., Maki D.G., Freebairn R.C., Kinasewitz G.T., Dhainaut J.F., Yan S.B., Williams M.D., Graham D.E. (2003). Systemic host responses in severe sepsis analyzed by causative microorganism and treatment effects of drotrecogin alfa (activated). Clin. Infect. Dis. Off. Publ. Infect. Dis. Soc. Am..

[10] Rauch U., Bonderman D., Bohrmann B., Badimon J.J., Himber J., Riederer M.A., Nemerson Y. (2000). Transfer of tissue factor from leukocytes to platelets is mediated by CD15 and tissue factor. Blood.

[11] Dellinger R.P., Levy M.M., Rhodes A., Annane D., Gerlach H., Opal S.M., Sevransky J.E., Sprung C.L., Douglas I.S., Jaeschke R. (2013). Surviving Sepsis Campaign: International guidelines for management of severe sepsis and septic shock, 2012. Intensive Care Med..

[12] Brun-Buisson C. (2000). The epidemiology of the systemic inflammatory response. Intensive Care Med..

[13] Hagiwara E., Abbasi F., Mor G., Ishigatsubo Y., Klinman D.M. (1995). Phenotype and frequency of cells secreting IL-2, IL-4, IL-6, IL-10, IFN and TNF-alpha in human peripheral blood. Cytokine.

[14] Levi M., Sivapalaratnam S. (2018). Disseminated intravascular coagulation: An update on pathogenesis and diagnosis. Expert Rev. Hematol..

[15] Niessen R.W., Lamping R.J., Jansen P.M., Prins M.H., Peters M., Taylor F.B., de Vijlder J.J., ten Cate J.W., Hack C.E., Sturk A. (1997). Antithrombin acts as a negative acute phase protein as established with studies on HepG2 cells and in baboons. Thromb. Haemost..

[16] Zhao C., Hu X., Bao L., Wu K., Feng L., Qiu M., Hao H., Fu Y., Zhang N. (2021). Aryl hydrocarbon receptor activation by Lactobacillus reuteri tryptophan metabolism alleviates Escherichia coli-induced mastitis in mice. PLoS Pathog..

[17] Choi H., Yang Z., Weisshaar J.C. (2017). Oxidative stress induced in E. coli by the human antimicrobial peptide LL-37. PLoS Pathog..

[18] Yu G.M., Tan W. (2019). Melatonin Inhibits Lipopolysaccharide-Induced Inflammation and Oxidative Stress in Cultured Mouse Mammary Tissue. Mediat. Inflamm..

[19] Murray J.D., Maga E.A. (2016). Genetically engineered livestock for agriculture: A generation after the first transgenic animal research conference. Transgenic Res..

[20] Robl J.M., Wang Z., Kasinathan P., Kuroiwa Y. (2007). Transgenic animal production and animal biotechnology. Theriogenology.

[21] Rogers C.S. (2016). Genetically engineered livestock for biomedical models. Transgenic Res..

[22] Brondyk W.H. (2009). Selecting an appropriate method for expressing a recombinant protein. Methods Enzymol..

[23] Keefer C.L. (2004). Production of bioproducts through the use of transgenic animal models. Anim. Reprod. Sci..

[24] Swanson M.E., Martin M.J., O’Donnell J.K., Hoover K., Lago W., Huntress V., Parsons C.T., Pinkert C.A., Pilder S., Logan J.S. (1992). Production of functional human hemoglobin in transgenic swine. Bio/technology.

[25] Sharma A., Martin M.J., Okabe J.F., Truglio R.A., Dhanjal N.K., Logan J.S., Kumar R. (1994). An isologous porcine promoter permits high level expression of human hemoglobin in transgenic swine. Bio/Technology.

[26] Zou X., Yuan S., Xian J., Zhao Y., Li D., Wang Y., Hao R., Jie J., Liu S., Du M. (2008). Large Scale Production of Recombinant Human Antithrombin Il(rhATI) in Transgenic Cloned Goats. Chin. J. Biotechnol..

[27] Hand T.W., Reboldi A. (2021). Production and Function of Immunoglobulin A. Annu. Rev. Immunol..

[28] Boes M., Prodeus A.P., Schmidt T., Carroll M.C., Chen J. (1998). A critical role of natural immunoglobulin M in immediate defense against systemic bacterial infection. J. Exp. Med..

[29] Roemisch J., Gray E., Hoffmann J.N., Wiedermann C.J. (2002). Antithrombin: A new look at the actions of a serine protease inhibitor. Blood Coagul. Fibrinolysis Int. J. Haemost. Thromb..

[30] Uchiba M., Okajima K., Murakami K., Okabe H., Takatsuki K. (1995). Effects of antithrombin III (AT III) and Trp49-modified AT III on plasma level of 6-keto-PGF1 alpha in rats. Thromb. Res..

[31] Yamauchi T., Umeda F., Inoguchi T., Nawata H. (1989). Antithrombin III stimulates prostacyclin production by cultured aortic endothelial cells. Biochem. Biophys. Res. Commun..

[32] Mizutani A., Okajima K., Uchiba M., Isobe H., Harada N., Mizutani S., Noguchi T. (2003). Antithrombin reduces ischemia/reperfusion-induced renal injury in rats by inhibiting leukocyte activation through promotion of prostacyclin production. Blood.

[33] Tateson J.E., Moncada S., Vane J.R. (1977). Effects of prostacyclin (PGX) on cyclic AMP concentrations in human platelets. Prostaglandins.

[34] Riva C.M., Morganroth M.L., Ljungman A.G., Schoeneich S.O., Marks R.M., Todd R.F., Ward P.A., Boxer L.A. (1990). Iloprost inhibits neutrophil-induced lung injury and neutrophil adherence to endothelial monolayers. Am. J. Respir. Cell Mol. Biol..

[35] Harada N., Okajima K., Kushimoto S., Isobe H., Tanaka K. (1999). Antithrombin reduces ischemia/reperfusion injury of rat liver by increasing the hepatic level of prostacyclin. Blood.

[36] Zhong K. (2015). Curcumin Mediates a Protective Effect Via TLR-4/NF-κB Signaling Pathway in Rat Model of Severe Acute Pancreatitis. Cell Biochem. Biophys..

[37] Wu W.H., Zegarra-Ruiz D.F., Diehl G.E. (2020). Intestinal Microbes in Autoimmune and Inflammatory Disease. Front. Immunol..

[38] Lima M.S.R., de Lima V.C.O., Piuvezam G., de Azevedo K.P.M., Maciel B.L.L., Morais A.H.A. (2022). Mechanisms of action of anti-inflammatory proteins and peptides with anti-TNF-alpha activity and their effects on the intestinal barrier: A systematic review. PLoS ONE.

[39] Mohammed Y., Kootte R.S., Kopatz W.F., Borchers C.H., Büller H.R., Versteeg H.H., Nieuwdorp M., van Mens T.E. (2020). The intestinal microbiome potentially affects thrombin generation in human subjects. J. Thromb. Haemost. JTH.

